# Comparing the diagnostic performance of ordinary, mixed, and lasso logistic regression models at identifying opioid and cannabinoid poisoning in U.S. dogs using pet demographic and clinical data reported to an animal poison control center (2005–2014)

**DOI:** 10.1371/journal.pone.0288339

**Published:** 2023-07-10

**Authors:** Mohammad Howard-Azzeh, David L. Pearl, Terri L. O’Sullivan, Olaf Berke

**Affiliations:** Department of Population Medicine, University of Guelph, Guelph, Ontario, Canada; Universidade Federal de Minas Gerais, BRAZIL

## Abstract

Researchers have begun studying the impact of human opioid and cannabinoid use on dog populations. These studies have used data from an animal poison control center (APCC) and there are concerns that due to the illicit nature and social stigma concerning the use of these drugs, owners may not always be forthcoming with veterinarians or APCC staff regarding pet exposures to these toxicants. As a result, models derived from APCC data that examine the predictability of opioid and cannabinoid dog poisonings using pet demographic and health disorder information may help veterinarians or APCC staff more reliably identify these toxicants when examining or responding to a call concerning a dog poisoned by an unknown toxicant. The fitting of epidemiologically informed statistical models has been useful for identifying factors associated with various health conditions and as predictive tools. However, machine learning, including lasso regression, has many useful features as predictive tools, including the ability to incorporate large numbers of independent variables. Consequently, the objectives of our study were: 1) identify pet demographic and health disorders associated with opioid and cannabinoid dog poisonings using ordinary and mixed logistic regression models; and 2) compare the predictive performance of these models to analogous lasso logistic regression models. Data were obtained from reports of dog poisoning events collected by the American Society for the Prevention of Cruelty to Animals’ (ASPCA) Animal Poisoning Control Center, from 2005–2014. We used ordinary and mixed logistic regression models as well as lasso logistic regression models with and without controlling for autocorrelation at the state level to train our models on half the dataset and test their predictive performance on the remainder. Although epidemiologically informed logistic regression models may require substantial knowledge of the disease systems being investigated, they had the same predictive abilities as lasso logistic regression models. All models had relatively high predictive parameters except for positive predictive values, due to the rare nature of calls concerning opioid and cannabinoid poisonings. Ordinary and mixed logistic regression models were also substantially more parsimonious than their lasso equivalents while still allowing for the epidemiological interpretation of model coefficients. Controlling for autocorrelation had little effect on the predictive performance of all models, but it did reduce the number of variables included in lasso models. Several disorder variables were associated with opioid and cannabinoid calls that were consistent with the acute effects of these toxicants. These models may help build diagnostic evidence concerning dog exposure to opioids and cannabinoids, saving time and resources when investigating these cases.

## Introduction

Drug-related death and abuse persist as a major public health concern, with opioids and cannabinoids amongst the most used drugs by humans in the USA [1]. Opioid-related human deaths continue to rise with over 65,000 in 2020 in the USA alone [2–4]. Similarly, the use and abuse of cannabinoids have increased following relaxed cannabis legislation [1, 5–7]. Despite the increasing severity of drug abuse in humans, little is known about the impact of these drugs on vulnerable populations, such as dogs.

Recent research has begun to unravel the impact of adult human opioid and cannabinoid use on dog populations [8–13]. These studies identified various risk factors such as income disparity, prescription rate, urbanicity, sex, weight, age, time, and reproductive status, that were associated with accidental dog poisonings [11, 12]. Previous work also examined the spatiotemporal distribution of opioid and cannabinoid poisoning events, identifying several space, time, and space-time clusters in the USA [13]. However, there have been no studies to examine the ability of data concerning clinical signs and dog characteristics reported by callers to predict toxicant type. Predictive models could be useful in aiding veterinarians and the public when the toxicant to which the dog was exposed is unknown or uncertain. Predictive models may be particularly useful to veterinarians when owners are hesitant to inform a veterinarian or the APCC that a dog was exposed to an opioid or cannabinoid due to the illicit nature and social stigma associated with the use of these drugs, helping to reduce delay in treatment or the need for additional diagnostic tests [11, 12]. These models may assist the APCC when diagnosing a potentially poisoned dog, which would be useful since the callers to APCC only have a high degree of certainty of the toxicant in approximately 38% of calls [14]. If these models can predict opioid and cannabinoid poisonings, predictive models may be used to predict other poisonings.

Statistical/epidemiological and algorithmic machine learning models have been created to predict opioid poisonings in humans, and have identified a number of variables associated with predicting opioid-related overdoses or deaths including: age, sex, race, socioeconomic status, urbanicity, mental and physical health comorbidities, substance use disorders, and type of opioid prescription [15–25]. These studies report high concordance statistics (c-statistic/area under the receiver operating characteristic curve), however, their practical application from a diagnostic perspective may be limited by their very low positive predictive values due to the rare occurrence of the different opioid-related outcomes investigated; in studies that include only people receiving a single opioid prescription, the opioid-related outcomes ranged from 0.05–0.49% of the study population [15, 26–31]. However, because of the high negative predictive values of these models (low proportion of false negatives) [26–31], they could be useful in preventing inappropriate interventions (e.g., providing naloxone for non-opioid related poisonings) [15]. Consequently, it may be useful to use available data concerning dog poisoning events to build predictive models for dog populations.

Several methods can be used to fit predictive models, with advantages and disadvantages for each. For binary outcomes (e.g., poisoned with an opioid vs. another toxicant), variables for a logistic regression model can be selected using an epidemiological approach. The main benefits of logistic regression models are that they can be built with a focus on causal reasoning, and the model coefficients can be converted to odds ratios and interpreted by epidemiologists to understand the strength and direction of the effect an independent variable has on the outcome. However, fitting epidemiologically informed models can be difficult with wide datasets and require researchers to have epidemiological and statistical training. Machine learning is a subset of artificial intelligence that creates mathematical models that can analyze and identify patterns in large datasets and use these patterns to make predictions. Machine learning has given rise to several automated algorithmic methods which can be used to build predictive models, including lasso regression models [32, 33]. Models, such as lasso regression, are substantially simpler to build if outcome prediction is the only objective and their ability to automatically select variables makes them particularly useful for datasets where a large number of independent variables must be considered. However, as lasso regression models are designed specifically for outcome prediction, the coefficients generated by lasso regression models are less meaningful and generally are not interpreted (i.e., the models are not typically used to understand the effect predictor variables have on the outcome) [34]. Additionally, the ability to adjust for autocorrelated data (e.g., the outcome an individual experiences is not independent from other members of a group, such as county or state of residence) is less developed for lasso regression models. Few methods exist to control for autocorrelation in lasso regression and few studies consider autocorrelation when using lasso regression to develop predictive models [35]. Currently, lasso regression models can only account for one level of clustering [35], which may limit their value when applied to data with multi-level structures (i.e., observations belonging to various groups within a hierarchical structure).

To date, no published studies have focused on examining the predictability of opioid or cannabinoid poisonings in dogs. This information and models that accurately predict opioid or cannabinoid poisonings would aid the public, veterinarians, and public health in identifying opioid or cannabinoid poisonings when the toxicant is unknown or not reported. It would also help to understand which clinical signs and animal characteristics that are most associated with opioid and cannabinoid poisoning. Comparing the predictive abilities between different predictive models would help understand which methods would be most suitable in diagnostic settings. The use of methods to control for autocorrelation are rarely used in lasso regressions, therefore examining the effect controlling for autocorrelation has on different predictive models would help understand the utility of such techniques and how they affect model performance. Therefore, the objectives of this study were the following: create predictive models using logistic regression models to examine their ability to predict opioid and cannabinoid poisoning events in US dogs using data from a national animal poison control center; identify disorders of body systems associated with these poisonings; compare the predictive ability of logistic regression models to models specifically designed for prediction (i.e., lasso logistic regression models); and examine the effect of controlling for autocorrelation on the performance of logistic and lasso logistic regression models.

## Methods

### Data

All the data used in this study were collected by the Animal Poison Control Center (APCC), which is operated by the American Society for the Prevention of Cruelty to Animals (ASPCA). The APCC provides over-the-phone emergency toxicological advice to the public, veterinarians, and other poison control centers that are administering care to a potentially poisoned animal. From each call, the APCC collects information concerning the number of animals exposed, toxicant, patient characteristics, clinical effects, outcome, and date/location/time of the call. These data are stored in the APCC’s AnTox toxicology database. The services for each case cost 65 USD at the time these data were gathered, and it is not mandatory for any party to make use of these services. Data from all 50 US states and the District of Columbia were used.

The maximum dataset used from the AnTox database during the study period included 217,495 unique observations. Each observation represents a potentially poisoned dog associated with a call reporting the event to the APCC between January 1, 2004 through December 31, 2014. The variables used in this study from each observation were dog-level characteristics: weight (kg), breed, age (years), reproductive status, sex, toxicant exposure, and the latitude/longitude of the call’s location (to identify state of the caller), as well as disorder category. The APCC categorized reported clinical signs in dogs into disorder categories. These disorder categories were recorded as present or absent and included the following: behavioural, digestive, cardiovascular, endocrine, general, hematopoietic, integumentary, lymphatic, metabolic, musculoskeletal, nervous, reproductive, respiratory, sensory, urinary, and traumatic disorders. The location data were used to identify the state of each call.

The data were analyzed for two outcomes, one concerning cannabinoid poisonings and another concerning opioid poisonings. For the analyses of cannabinoid poisonings, a cannabinoid call was defined as any call to the APCC concerning a dog that was exposed to any form of cannabinoid or cannabinoid derivative including: raw cannabis regardless of species, tetrahydrocannabinol, cannabidiol, synthetic cannabinoids, prescription cannabis, cannabis oils, and hemp seed oil. Cannabinoid products were often present in edible foods, such as brownies. If a dog was exposed to a cannabinoid product and another toxicant at the time of the call to the APCC, it was also considered a cannabis call. A non-cannabis call was defined as a call to the APCC regarding a dog that was only exposed to non-cannabinoid-related toxicants.

An opioid call was defined as any call to the APCC from a dog exposed to at least one type of opioid product. This included all prescription and illicit opioids as well as over-the-counter drugs containing opioids that could be abused. If a dog was exposed to an opioid and another toxicant when the call was made to the APCC, it was considered an opioid call. A non-opioid call was considered any call to the APCC from a dog that was only exposed to non-opioid toxicants.

The categorization of some variables has been changed from their original classification in the AnTox database. The reproductive status variable was originally coded as immature, intact, lactating, neutered, pregnant, or unknown. These data were used to determine if animals were intact, neutered, or unknown for subsequent analyses. The original AnTox coding of the sex variable was male, female, did not ask, group, and unknown. These data were used to reclassify the dogs for this study as female, male, or unknown.

The AnTox database contained information regarding each dog’s primary/apparent breed. These data were used to assign each dog to the following American Kennel Club (AKC) breed classes: herding, hound, non-sporting, sporting, terrier, toy, working, Foundation Stock Service (FSS), and other. Dogs whose breeds fell under AKC’s miscellaneous category (n = 40) were re-classified as part of the FSS category. Dog breeds that are not recognized by the AKC were classified into the “other” category. When the data were randomly divided into training and testing datasets, all cannabinoid poisoning calls for the "FSS & miscellaneous" breed class category ended up in the testing dataset. Therefore, for the cannabinoid models, the "other" breed class was combined with the "FSS & miscellaneous" breed class and randomly selected again to make certain the same variables were present in the training and testing datasets. The AnTox database contained a field for describing each dog’s breed as mixed, pure, or if the owners were not asked. Approximately 74% of the observations had the field marked as “not asked”, therefore the purity of the breed was not considered and only the primary/apparent breed was used to classify the breed class of each dog.

Observations for the weight and age variables with impossible values recorded were treated as missing data. Weights recorded as “0” (n = 1,698) or exceeding 114 kg for giant breed dogs (Great Danes, Mastiffs, Neapolitan Mastiffs, Tibetan Mastiffs, Leonbergers, Boerboels, Newfoundlands, St. Bernards) (n = 0) or exceeding 75 kg for all other breeds were not used in this study (n = 37). Ages recorded as “0” (n = 1,816) or greater than 26 years old (n = 13) were not used in this study.

Regarding the use of the phrase "statistically significant" [36], in this manuscript, the term "statistically significant" is not intended to infer biological/epidemiological importance or causation. It is used to indicate that based on our statistical criteria, we have enough evidence to infer that the measure of association for a given predictor variable or contrast is different from the null value [11]. The term “statistical significance” was used to describe results in an exploratory sense since our study involved a pre-existing dataset [37].

### Analyses

**Fig 1:**
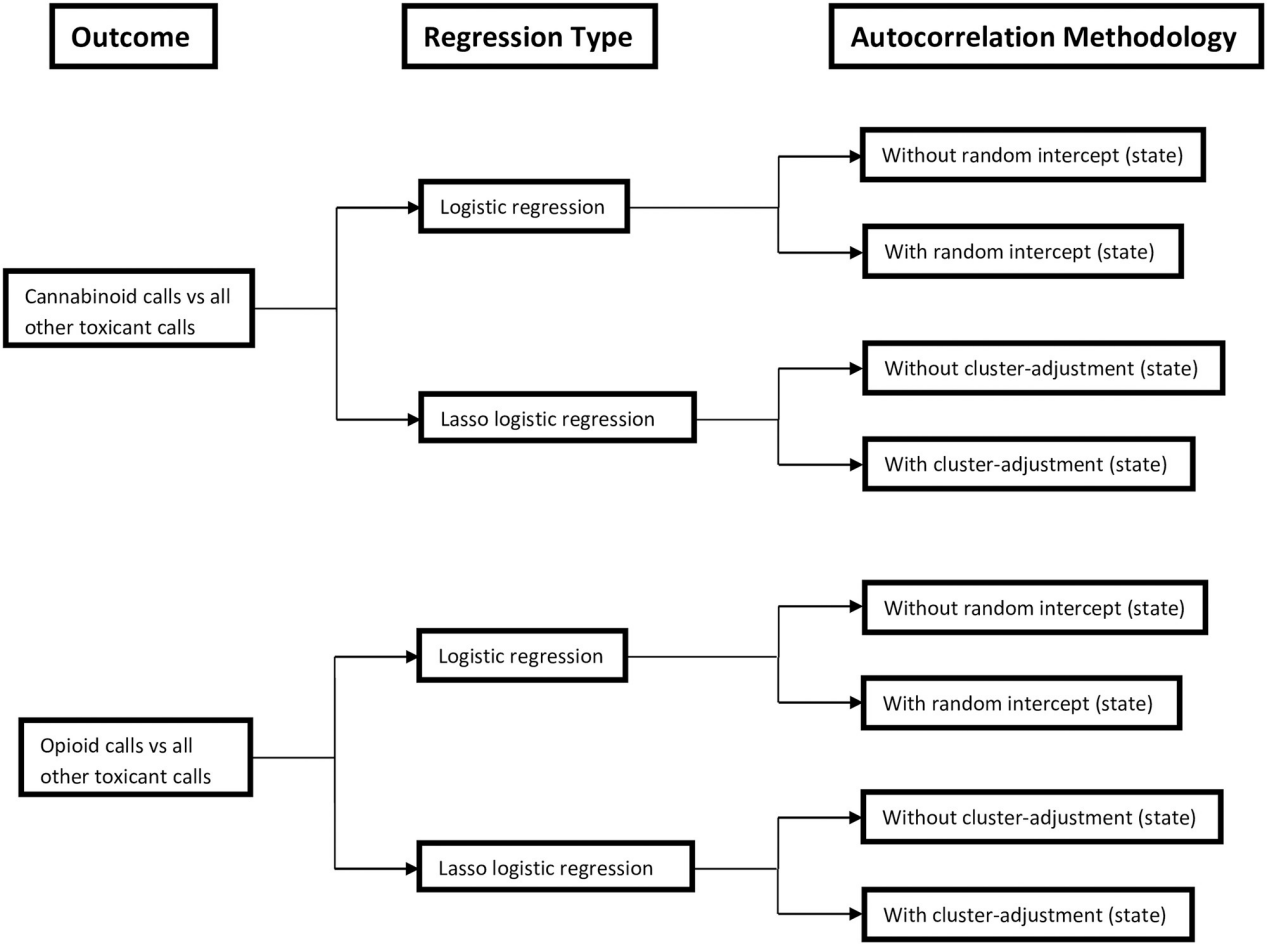
Flow chart depicting the 8 different logistic regression models fitted to compare their ability to predict opioid and cannabinoid poisoning calls to the APCC^a^ in US dogs (2005–2014).

This study used four regression models to compare their ability to predict a cannabinoid or opioid poisoning from the AnTox dataset. For each toxicant, an ordinary logistic regression, a logistic regression with a random intercept for state, a lasso logistic regression, and a lasso logistic regression that adjusted for clustering by state [35] were fitted (Fig 1). The data were randomly divided into two datasets: a training dataset, used to build the models; and a testing dataset, used to evaluate each model’s predictive ability. It was only possible to account for clustered data in lasso regressions at one level. Therefore, to make models comparable, we only controlled for clustering at the state level in lasso and mixed logistic regressions. Households reporting multiple dog calls were used in this study. The data in this study were analyzed using Stata 17 (StataCorp, College Station, TX).

To assess a model’s predictive ability, the sensitivity, specificity, positive predictive value, negative predictive value, area under the receiver operating characteristic curve (concordance statistic), the proportion of correctly classified cases, and deviance ratios were reported. When building predictive models with rare outcomes (i.e., less than 5% of observations), models will favour cut-points with 100% specificity and 0% sensitivity to achieve the highest correctly identified proportions; this situation is also referred to as a class imbalance. Therefore, probability cut-points were chosen where sensitivity versus probability and specificity versus probability curves intersected to optimize the balance between sensitivity and specificity [38]. Odds ratios, confidence intervals, and p-values are reported for ordinary and mixed logistic regression models. All variables used in the final lasso regression models are reported with their respective coefficients. However, coefficients estimated with lasso regression do not have confidence intervals or p-values and should not be interpreted [39].

### Ordinary and mixed logistic regression modeling process

Descriptive statistics, including frequencies, means, medians, interquartile ranges, and standard deviations, were estimated for all independent variables. All descriptive statistics were reported based on the type of data (i.e., continuous or dichotomous) used for subsequent modelling. The correlation between independent variables was examined using various correlation coefficients (i.e., Pearson, Phi, and Spearman’s rank) depending on the type of independent variables. If the correlation between two variables was greater than |0.75|, the more epidemiologically plausible variable was kept in the model moving forward. Locally weighted scatterplot smoothing (LOWESS) curves were used to assess the relationship between the continuous independent variables and the log odds of being a cannabinoid or opioid-related call. If the relationship was linear, the variable was left unchanged, if the relationship was not linear, the independent variable was categorized, or if appropriate, was modelled as a quadratic relationship with the addition of a squared term to the model (e.g., age^2^).

Ordinary univariable logistic regression and mixed univariable logistic regression models were fitted to assess the association between the independent variables and the log odds of a dog poisoning being related to either an opioid or a cannabinoid. Independent variables with significant associations (α = 0.05) were considered for inclusion in their respective multivariable models. All mixed models (i.e., univariable and multivariable) included a random intercept for state.

**Fig 2:**
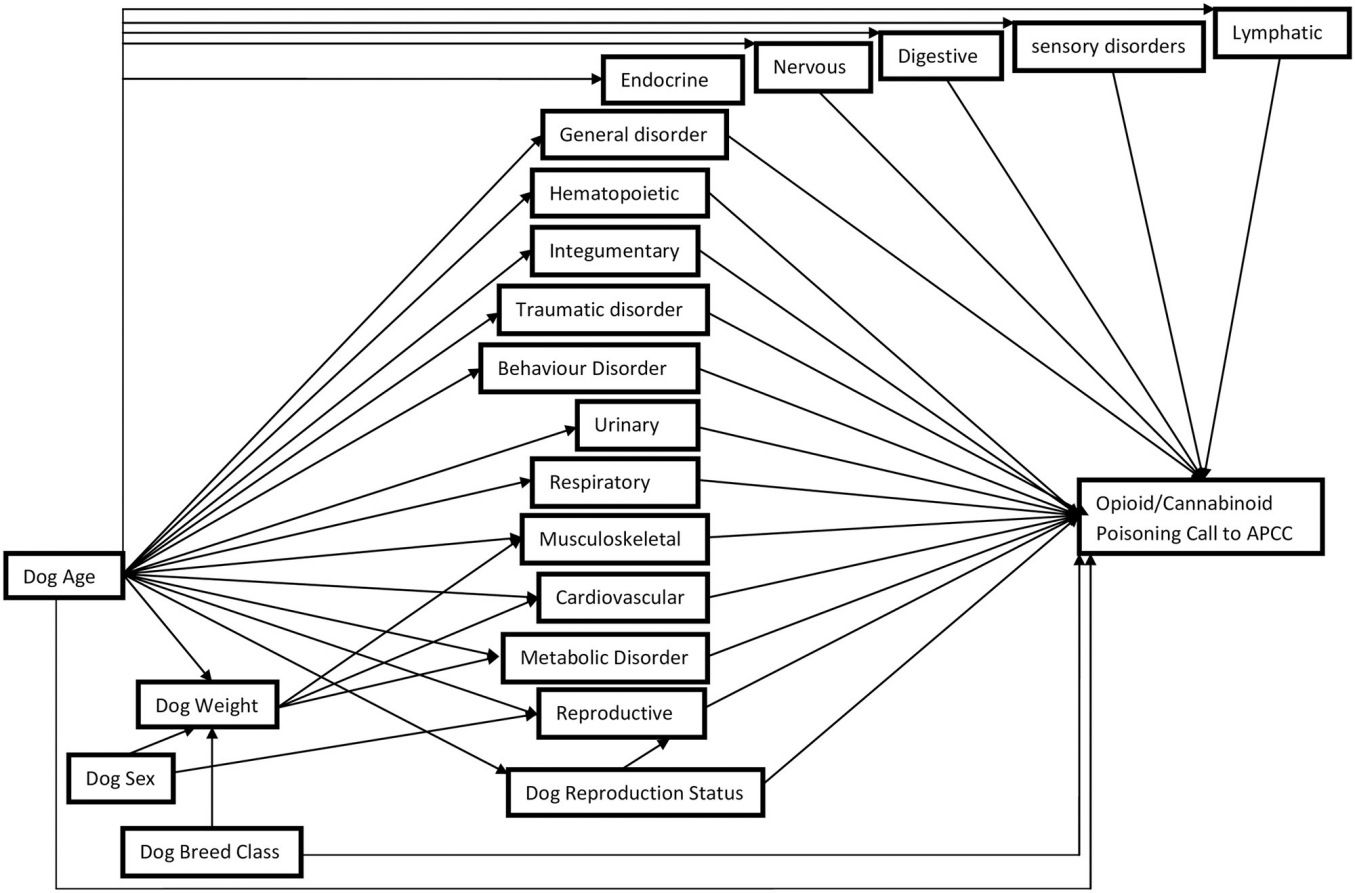
Causal diagram depicting the relationship between dog characteristics and disorder type and calls being related to an intoxication with a cannabinoid or opioid.

Manual backward variable selection was performed in the multivariable regression model building process. Independent variables were removed one at a time, starting with those with the highest p-values (lowest Wald’s χ^2^ values) until all variables met the statistical criteria. Independent variables were included in the final multivariable models if they were statistically significant (α = 0.05), affected the coefficients of a significant independent variable as an explanatory antecedent or distorter, or were part of a statistically significant interaction. Independent variables that did not meet the statistical criteria in the backward model building process were re-introduced to the model one at a time. If a re-introduced variable caused a 20% change or greater in the coefficient of any significant variable when re-introduced, it was considered an explanatory antecedent (i.e., confounder if effect reduced) or distorter variable (i.e., effect increased or direction of association changed), given it met the causal criteria (i.e., non-intervening variable) based on the causal diagram (Fig 2). Biologically relevant two-way interactions (all two-way interactions between weight, age, sex, breed class, and reproductive status) that were identified *a priori* were assessed one at a time in the main effects model.

The fit of ordinary logistic regression models was assessed using a Hosmer-Lemeshow goodness-of-fit test. Pearson and deviance residuals were assessed to identify outliers. For mixed logistic regression models, the normality and homoscedasticity assumptions for the random effect (i.e., best linear unbiased predictors (BLUPs)) were assessed graphically using normal quantile plots and plotting the BLUPs against the predicted outcome, respectively. Pearson residuals were assessed to identify outliers. Variance partition coefficients at the dog and state levels were estimated from the variance components from the final model using the latent variable technique [40].

### Lasso regression modeling process

One of the goals of this study was to compare methods used by epidemiologists to fit epidemiologically-informed logistic regression models using a causal diagram to lasso regression that can be readily automated to deal with issues concerning variable selection. With automation in mind, collinearity among independent variables and linearity between the independent variables and the outcome were not assessed. All possible independent variables of interest in our dataset (Fig 2), including quadratic terms of continuous variables (e.g., age^2^), and all possible two-way interactions were available for selection in our lasso logistic regression models using 10-fold cross-validation. For models intended to account for clustered data, we used the cluster plug-in cross-validation method to split observations by cluster group (i.e., by state) where the subsample is drawn in each fold by cluster group [35]. Lasso regression algorithms pre-process potential continuous variables in the model by standardizing the variables mean to 0 and their standard deviation to 1 [39].

## Results

### Descriptive statistics

As described in Howard-Azzeh et al. (2020, 2021, and 2022), cannabinoid and opioid poisonings made up 0.98% (n = 2,133) and 2.74% (n = 5,962) of all poisoning calls (n = 217,495) to the APCC, respectively. Dogs in this dataset were relatively small and young, with median ages of 2 years and a median weight of 12.2 kg (Table 1). Female dogs represented slightly more poisoning calls in this dataset than male dogs (Table 2). Of all dogs in this dataset, 74.30% were neutered and 22.07% were intact (Table 2). Toy dogs were the largest breed class (24.22%), while there were very few observations from the FSS breed class (0.27%) (Table 2).

**Table 1:** Descriptive statistics concerning the age and weight of dogs from US calls reporting poisoning events to the APCC^a^ (2005–2014).

Parameter	Mean	Median	Standard Deviation	Interquartile Range	N^‡^
**Age (Years)**	3.6	2.0	3.5	0.9–6	215,589
**Weight (kg)**	16.4	12.2	12.7	5.8–25.8	215,684

^‡^N identifies the number of dog-associated calls in the dataset with these variables

**Table 2:** The sex, breed, and neuter status of dogs from US calls reporting poisoning events to the APCC^a^ (2005–2014).

Parameter	Frequency	Percentage of dataset
**Sex**	N = 217,495	
Female	111,830	51.42
Male	104,649	48.12
Unknown	1,016	0.47
**Breed Class** ^b^	N = 217,495	
Herding	18,543	8.50
Hound	19,663	9.04
Foundation Stock Service	586	0.27
Non-Sporting	18,136	8.34
Sporting	50,911	23.41
Terrier	23,619	10.86
Toy	52,685	24.22
Working	19,167	8.81
Other^c^	14,185	6.52
**Reproductive Status**	N = 217,495	
Intact	48,002	22.07
Neutered	161,605	74.30
Unknown	7,888	3.63

^a^Animal Poison Control Center

^b^Breed classes as defined by the American Kennel Club based on the primary breed reported

^c^Breeds in the AnTox database that are not yet categorized into American Kennel Club breed classes

N identifies the number of dog-associated calls in the dataset with these variables

Most disorder categories were reported relatively frequently (i.e., >4000 dogs). However, endocrine, lymphatic, reproductive, and traumatic disorders were reported infrequently, making up 0.01% (n = 27), 0.07% (n = 148), 0.05% (n = 111), and 0.06% (n = 128) of all poisoning calls, respectively (Table 3).

**Table 3:** The frequency of disorders in US dogs reported to the APCC^a^ (2005–2014).

Parameter	Frequency	Percentage of dataset
**Behavioural Disorders**	N = 217,415	
Not Reported	186,468	85.77
Reported	30,947	14.23
**Digestive Disorders**	N = 217,415	
Not Reported	85,422	39.29
Reported	131,993	60.71
**Cardiovascular Disorders**	N = 217,415	
Not Reported	190,658	87.69
Reported	26,757	12.31
**Endocrine Disorders**	N = 217,415	
Not Reported	217,388	99.99
Reported	27	0.01
**General Disorders**	N = 217,415	
Not Reported	167,819	77.19
Reported	49,596	22.81
**Hematopoietic Disorders**	N = 217,415	
Not Reported	212,588	97.78
Reported	4,827	2.22
**Integumentary Disorders**	N = 217,415	
Not Reported	211,588	97.32
Reported	5,827	2.68
**Lymphatic Disorders**	N = 217,415	
Not Reported	217,267	99.93
Reported	148	0.07
**Metabolic Disorders**	N = 217,415	
Not Reported	209,034	96.15
Reported	8,381	3.85
**Nervous Disorders**	N = 217,415	
Not Reported	130,206	59.89
Reported	87,209	40.11
**Reproductive Disorders**	N = 217,415	
Not Reported	217,304	99.95
Reported	111	0.05
**Respiratory Disorders**	N = 217,415	
Not Reported	196,878	90.55
Reported	20,537	9.45
**Sensory Disorders**	N = 217,415	
Not Reported	204,870	94.23
Reported	12,545	5.77
**Urinary Disorders**	N = 217,415	
Not Reported	201,141	92.51
Reported	16,274	7.49
**Traumatic Disorders**	N = 217,415	
Not Reported	217,287	99.94
Reported	128	0.06
**Musculoskeletal Disorders**	N = 217,415	
Not Reported	208,324	95.82
Reported	9,091	4.18

^a^Animal Poison Control Center

N identifies the number of dog-associated calls in the dataset with these variables

### Number of variables included

For both cannabinoid and opioid calls, the models fit with lasso logistic regression had the largest number of coefficients followed by models fit with cluster-adjusted lasso logistic regression (Table 4). Ordinary and mixed logistic regression models were fit with the fewest coefficients for both cannabinoid and opioid calls (Table 4).

**Table 4:** Number of coefficients in models fitted using various logistic regression models examining the associations between dog-level variables and a poisoning call to the APCC^a^ being related to cannabinoids or opioids (2005–2014).

Model (Regression)	Number of Coefficients
**Cannabinoid Lasso Logistic**	117
**Cluster-Adjusted Cannabinoid Lasso Logistic**	46
**Cannabinoid Logistic**	22
**Cannabinoid Logistic with Random Intercept (state)**	22
**Opioid Lasso Logistic**	169
**Cluster-Adjusted Opioid Lasso Logistic**	45
**Opioid Logistic**	27
**Opioid Logistic with Random Intercept (state)**	27

^a^Animal Poison Control Center

### Predictive ability

The predictive abilities of all cannabinoid models were almost identical (Table 5). Across all four models, sensitivity and specificity ranged from 76.4–77.0% and 76.5–78.0%, respectively. The positive predictive values were very small, ranging from 3.1–3.3%. Negative predictive values were the same for all four models at 97.7%. The percent correctly classified ranged from 76.5–78.0%.

**Table 5:** Statistics concerning different logistic regression models’ abilities to predict cannabinoid poisoning calls to the APCC^a^ in US dogs (2005–2014).

Method	Sensitivity (95% CIs)	Specificity (95% CIs)	Positive predictive value (95% CIs)	Negative predictive value (95% CIs)	ROC area^b^ (95% CIs)	Correctly Classified
**Logistic Regression**	76.4% (73.7; 78.9)	76.5% (76.2; 76.7)	3.1% (2.9; 3.3)	99.7% (99.7; 99.7)	85.2% (84.4; 86.1)	76.5% (76.2; 76.7)
**Logistic Regression with Random Intercept (State)**	76.9% (76.6; 77.1)	78.0% (77.8; 78.3)	3.3% (3.2; 3.6)	99.7% (99.7; 99.7)	86.6% (85.7; 87.4)	78.0% (77.7; 78.2)
**Lasso Logistic Regression**	76.7% (74.0; 79.3)	77.0% (76.7; 77.2)	3.2% (3.0; 3.4)	99.7% (99.7; 99.7)	85.7% (84.8; 86.5)	77.0% (76.7; 77.2)
**Cluster-Adjusted Lasso Logistic Regression (State)**	77.0% (74.3; 79.5)	77.1% (76.8; 77.3)	3.2% (3.0; 3.4)	99.7% (99.7; 99.7)	84.8% (83.9; 85.7)	77.1% (76.8; 77.3)

^a^Animal Poison Control Center

^b^ Area under the receiver operating characteristic curve (concordance statistic)

Like cannabinoid models, the predictive abilities of opioid models were also very similar in all four models (Table 6). The sensitivity and specificity ranged from 65.9–67.3% and 66.0–67.2%, respectively. The positive predictive values were small, ranging from 5.1–5.4%. Negative predictive values ranged from 98.6–98.7%. The percent of the outcome correctly classified ranged from 66.0–67.2%.

**Table 6:** Statistics concerning different logistic regression models’ abilities to predict opioid poisoning calls to the APCC^a^ in US dogs (2005–2014).

Method	Sensitivity (95% CIs)	Specificity (95% CIs)	Positive predictive value (95% CIs)	Negative predictive value (95% CIs)	ROC area^b^ (95% CIs)	Correctly Classified
**Logistic Regression**	67.0% (65.2; 68.7)	67.0% (66.7; 67.3)	5.4% (5.1; 5.6)	98.6% (98.6; 98.7)	74.2% (72.8; 75.7)	67.0% (66.7; 67.3)
**Logistic Regression with Random Intercept (State)**	66.8% (68.8; 67.2)	67.2% (66.9; 67.4)	5.4% (5.2; 5.5)	98.6% (98.6; 98.7)	73.6% (72.2; 75.1)	67.2% (66.9; 67.4)
**Lasso Regression**	65.9% (64.2; 67.6)	66.0% (65.7; 66.3)	5.1% (4.9; 5.3)	98.6% (98.5; 98.7)	74.0% (73.1; 74.9)	66.0% (65.7; 66.2)
**Cluster-Adjusted Lasso Regression (State)**	67.3% (65.5; 69.0)	67.2% (66.9; 67.5)	5.4% (5.2; 5.6)	98.7% (98.6; 98.7)	71.0% (70.1; 71.9)	67.2% (66.9; 67.5)

^a^Animal Poison Control Center

^b^ Area under the receiver operating characteristic curve (concordance statistic)

For cannabinoid and opioid models, the deviance ratios from the testing dataset were higher for ordinary logistic regression models than lasso regression models (Tables 7 and 8). There was also little difference in the deviance ratios between the training and the testing datasets for ordinary logistic regression models. The difference in deviance ratios between the training and the testing datasets was generally much larger for lasso regression models.

**Table 7:** Deviance ratios depicting different logistic regression models’ abilities to predict cannabinoid poisoning calls to the APCC^a^ in US dogs from their respective training and testing datasets (2005–2014).

Model	Sample	Deviance	Deviance Ratio
**Logistic Regression**	Training	0.0907	0.1790
	Testing	0.0906	0.1767
**Logistic Regression with Random Intercept (state)**	Training	N/A*	N/A*
	Testing	N/A*	N/A*
**Lasso Regression**	Training	0.08907	0.1923
	Testing	0.09367	0.1427
**Cluster-Adjusted Lasso Regression (State)**	Training	0.09418	0.1460
	Testing	0.09468	0.1334

^a^Animal Poison Control Center

*Not available

**Table 8:** Deviance ratios depicting different logistic regression models’ abilities to predict opioid poisoning calls to the APCC^a^ in US dogs from their respective training and testing datasets (2005–2014).

Model	Sample	Deviance	Deviance Ratio
**Logistic Regression**	Training	0.2310	0.0881
	Testing	0.2272	0.0883
**Logistic Regression with Random Intercept (state)**	Training	N/A*	N/A*
	Testing	N/A*	N/A*
**Lasso Regression**	Training	0.2291	0.0940
	Testing	0.2344	0.0586
**Cluster-Adjusted Lasso Regression (State)**	Training	0.2379	0.0592
	Testing	0.2358	0.0529

^a^Animal Poison Control Center

*Not available

### Variables included

#### Cannabinoid models

The variables used in each of the models varied substantially. The following variables were included in both ordinary and mixed cannabinoid logistic regression models: age, age^2^, sex, breed class, digestive disorders, cardiovascular disorders, hematopoietic disorders, integumentary disorders, metabolic disorders, nervous disorders, respiratory disorders, sensory disorders, and urinary disorders (Table 9). Of these variables, only weight, age^2^, sporting breed class, digestive disorders, general disorders, integumentary disorders, metabolic disorders, nervous disorders, and sensory disorders were fitted into the lasso logistic regression as main effects (S1 Table). The remaining 99 coefficients were interaction terms, in which most of their respective main effects were not included in the model. For the cluster-adjusted lasso logistic regression, digestive and nervous disorders were the only main effects that were included in the model. The remaining 44 coefficients were interaction terms where most of the interaction terms did not have their main effect included in the model (S2 Table).

**Table 9:** Results of ordinary and mixed multivariable logistic regression models examining the associations between each dog-level variable on the odds of a poisoning call to the APCC^a^ being related to cannabinoids (2005–2014).

Cannabinoid Logistic regression				Cannabis Logistic Regression With Random Intercept (state)		
Parameter	OR	95% CIs	P-value	OR	95% CIs	P-value
**Age**	1.07	1.001; 1.13	0.027	1.06	0.998; 1.12	0.058
**Age** ^**2**^	0.99	0.99; 0.999	0.012	0.995	0.99; 0.999	0.025
**Sex**						
Female	Referent			Referent		
Male	1.20	1.06; 1.36	0.004	1.20	1.06; 1.35	0.005
Unknown	0.72	0.18; 2.95	0.651	0.79	0.19; 3.24	0.746
**Breed Class**						
Herding	Referent			Referent		
Hound	0.85	0.61; 1.17	0.311	0.85	0.62; 1.18	0.329
FSS, Misc & Other	1.17	0.85; 1.17	0.330	1.18	0.86; 1.63	0.314
Non-Sporting	1.31	0.97; 1.77	0.076	1.26	0.93; 1.71	0.130
Sporting	0.80	0.61; 1.05	0.108	0.79	0.60; 1.04	0.098
Terrier	1.19	0.89; 1.58	0.234	1.18	0.88; 1.57	0.269
Toy	1.34	1.04; 1.72	0.022	1.29	1.004; 1.66	0.046
Working	0.93	0.67; 1.28	0.646	0.92	0.67; 1.27	0.614
**Digestive**						
Not Reported	Referent			Referent		
Reported	0.45	0.40; 0.52	<0.001	0.45	0.39; 0.52	<0.001
**Cardiovascular**						
Not Reported	Referent			Referent		
Reported	1.51	1.30; 1.76	<0.001	1.50	1.29; 1.75	<0.001
**General**						
Not Reported	Referent			Referent		
Reported	1.29	1.130; 1.47	<0.001	1.31	1.15; 1.49	<0.001
**Hematopoietic**						
Not Reported	Referent			Referent		
Reported	0.27	0.13; 0.58	0.001	0.27	0.13; 0.58	0.001
**Integumentary**						
Not Reported	Referent			Referent		
Reported	0.11	0.035; 0.34	<0.001	0.11	0.035; 0.34	<0.001
**Metabolic**						
Not Reported	Referent			Referent		
Reported	0.44	0.29; 0.68	<0.001	0.45	0.29; 0.68	<0.001
**Nervous**						
Not Reported	Referent			Referent		
Reported	20.28	15.54; 26.47	<0.001	20.67	15.83; 26.97	<0.001
**Respiratory**						
Not Reported	Referent			Referent		
Reported	0.23	0.16; 0.33	<0.001	0.23	0.16; 0.33	<0.001
**Sensory Disorder**						
Not Reported	Referent			Referent		
Reported	1.80	1.52; 2.14	<0.001	1.83	1.54; 2.17	<0.001
**Urinary**						
Not Reported	Referent			Referent		
Reported	3.09	2.58; 3.69	<0.001	3.00	2.51; 3.59	<0.001
**Variance Component (State)**				0.17	0.084; 0.34	

^a^Animal Poison Control Center

#### Opioid models

Like the cannabinoid models, the variables used in each of the models varied substantially. The following variables were included in both ordinary and mixed opioid logistic regression models: weight, weight^2^, age, age^2^, weight X age interaction, sex, reproductive status, breed class, behavioural disorders, digestive disorders, cardiovascular disorders, general disorders, hematopoietic disorders, integumentary disorders, metabolic disorders, nervous disorders, respiratory disorders, and sensory disorders (Table 10). In the lasso logistic regression, the main effects included in the model were: age, age^2^, weight, "unknown" sex, FSS & miscellaneous breed class, sporting breed class, toy breed class, intact reproductive status, general disorders, hematopoietic disorders, integumentary disorders, metabolic disorders, nervous disorders, reproductive disorders, urinary disorders, traumatic disorders (S3 Table). The remaining 153 coefficients were interaction terms, in which most of their main effects were not included in the model. In the cluster-adjusted lasso logistic regression, the main effects included in the model were: age, weight, "unknown" sex, digestive disorders, general disorders, hematopoietic disorders, integumentary disorders, metabolic disorders, nervous disorders, urinary disorders (S4 Table). The remaining 35 coefficients were interaction terms, and most of their main effects were also not in the model.

**Table 10:** Results of ordinary and mixed multivariable logistic regression models examining the associations between each dog-level variable on the odds of a poisoning call to the APCC^a^ being related to opioids (2005–2014).

	Opioid Logistic Regression			Opioid Logistic With Random Intercept (state)		
Parameter	OR	95% CIs	P-value	OR	95% CIs	P-value
**Weight**	0.98	0.97; 0.99	0.001	0.98	0.97; 0.99	0.001
**Weight** ^**2**^	1.0003	1.00008; 1.0005	0.006	1.0003	1.00009; 1.0005	0.006
**Age**	0.79	0.76; 0.82	<0.001	0.79	0.76; 0.83	<0.001
**Age** ^**2**^	1.01	1.01; 1.02	<0.001	1.01	1.01; 1.02	<0.001
**Weight x Age**	1.001	1.00001; 1.002	0.047	1.001	1.00001; 1.002	0.048
**Sex**						
Female	Referent					
Male	0.92	0.85; 0.99	0.025	0.92	0.85; 0.99	0.028
Unknown	0.89	0.43; 1.83	0.741	0.88	0.43; 1.83	0.736
**Reproductive Status**						
Intact	Referent					
Neutered	0.88	0.80; 0.97	0.007	0.88	0.81; 0.97	0.009
Unknown	0.88	0.70; 1.09	0.241	0.88	0.71; 1.10	0.265
**Breed Class**						
Herding	Referent			Referent		
Hound	1.32	1.10; 1.60	0.004	1.31	1.09; 1.59	0.005
FSS & Misc	0.44	0.14; 1.39	0.162	0.44	0.14; 1.39	0.161
Non-Sporting	1.06	0.87; 1.30	0.549	1.06	0.87; 1.30	0.563
Sporting	0.82	0.70; 0.98	0.025	0.82	0.69; 0.97	0.020
Terrier	1.29	1.08; 1.55	0.005	1.29	1.08; 1.54	0.006
Toy	1.43	1.19; 1.70	<0.001	1.42	1.19; 1.70	<0.001
Working	0.88	0.72; 1.08	0.23	0.88	0.71; 1.08	0.205
Other	0.92	0.74; 1.14	0.417	0.92	0.74; 1.14	0.419
**Behavioural**						
Not Reported	Referent			Referent		
Reported	0.74	0.66; 0.83	<0.001	0.74	0.69; 0.83	<0.001
**Digestive**						
Not Reported	Referent			Referent		
Reported	0.83	0.77; 0.90	<0.001	0.84	0.77; 0.90	<0.001
**Cardiovascular**						
Not Reported	Referent			Referent		
Reported	1.27	1.15; 1.41	<0.001	1.27	1.15; 1.41	<0.001
**General**						
Not Reported	Referent			Referent		
Reported	1.48	1.36; 1.60	<0.001	1.48	1.36; 1.61	<0.001
**Hematopoietic**						
Not Reported	Referent			Referent		
Reported	0.45	0.30; 0.69	<0.001	0.45	0.30; 0.69	<0.001
**Integumentary**						
Not Reported	Referent			Referent		
Reported	0.21	0.13; 0.34	<0.001	0.21	0.13; 0.34	<0.001
**Metabolic**						
Not Reported	Referent			Referent		
Reported	0.33	0.25; 0.45	<0.001	0.33	0.25; 0.45	<0.001
**Nervous**						
Not Reported	Referent			Referent		
Reported	2.69	2.48; 2.92	<0.001	2.69	2.48; 2.92	<0.001
**Respiratory**						
Not Reported	Referent			Referent		
Reported	1.21	1.07; 1.36	0.002	1.21	1.07; 1.36	0.002
**Sensory**						
Not Reported	Referent			Referent		
Reported	0.41	0.33; 0.51	<0.001	0.41	0.33; 0.51	<0.001
Variance Component (State)				0.021	0.0091; 0.049	

^a^Animal Poison Control Center

### Interpretation of ordinary and mixed logistic regression coefficients

#### Cannabinoid models

The logistic regression models identified several statistically significant relationships that can be readily interpreted. There was a statistically significant relationship between age and the odds of a poisoning call being related to a cannabinoid (Table 9). The predicted probability of a cannabinoid poisoning call initially increased with age and declined after 5 years (S1 Fig). The odds of a dog cannabinoid call were significantly greater for male dogs than female dogs and varied among breeds (Table 8).

A cannabinoid poisoning call had a greater odds of occurring when a cardiovascular, general, nervous, sensory, or urinary disorder was reported than not reported. A cannabinoid poisoning call had a reduced odds of occurring when a digestive, hematopoietic, integumentary, metabolic, or respiratory disorder was reported than not reported (Table 8).

The variance partition coefficients indicate that approximately 95.2% and 4.9% of the variance was explained at the dog and state levels, respectively (Table 8).

#### Opioid models

As age increased, the odds of an opioid poisoning call decreased, however this effect seemed to plateau around six years old (S2 Fig). For the youngest dogs, the odds of an opioid poisoning call initially decreased with weight, but then flattened for heavier dogs. For older dogs, the effect of weight was less pronounced, and the odds of an opioid poisoning call did not change substantially as weight increased (S2 Fig). Female and intact dogs had a greater odds of an opioid poisoning call than male and neutered dogs, respectively, and varied among breeds (Table 10).

An opioid poisoning call had a significantly greater risk of occurring when a cardiovascular, general, nervous, or a respiratory disorder category was reported than not reported. An opioid poisoning call had a significantly reduced risk of occurring when a behavioural, digestive, hematopoietic, integumentary, metabolic, or sensory disorder category was reported than not reported (Table 10).

The variance partition coefficients indicate that approximately 99.4% and 0.6% of the variance was explained at the dog and state levels, respectively (Table 10).

## Discussion

This study examined the predictive ability of models for opioid and cannabinoid poisonings in dogs throughout the US based on reported poisoning events, potentially aiding in the identification of opioid and cannabinoid poisonings when the toxicant is unknown or not reported. Using call data provided by the ASPCA concerning dog poisonings reported to the APCC, we fit logistic regression and lasso logistic regression models and assessed their ability to predict opioid or cannabinoid poisonings in dogs. We also examined the influence of controlling for autocorrelation on the models’ performance and examined the associations between reported dog disorder categories and dog cannabinoid and opioid poisoning events from logistic regression models.

### Predictive ability

The predictive performance of the models fit in this study were similar regardless of the model building strategy; there were no substantial differences in the predictive abilities between logistic or lasso logistic regression models, with and without controlling for autocorrelation at the state level. The predictor variables available from the AnTox dataset used to fit our models had low positive predictive values for opioid and cannabinoid poisonings regardless of modeling approach. This is consistent with predictive models aimed at predicting opioid outcomes created from human data [15–25]. It is possible that if many more or more predictive predictor variables (e.g., more clinical signs and test results) were available, more of a difference would have been observed between the modeling approaches and the lasso logistic regression models would have outperformed the logistic regression models [41]. It is important to note that since the outcome is rare (opioid and cannabinoid poisoning calls), these data are considered class imbalanced. Although we dealt with class imbalance by adjusting cut-off values to optimize sensitivity and specificity using a receiver operating characteristic curve approach, there are several other methods to adjust for class imbalance that might have resulted in different model performances [38]. Based on our findings, we recommend that the decision to choose between a logistic or lasso logistic regression when fitting a model depends on the researcher’s needs and abilities. A lasso logistic regression may best be used when the researcher’s goals are purely predictive and making inferences from estimated odds ratios is not necessary. Lasso logistic regressions are particularly useful when building predictive models based on very wide datasets to take advantage of automated variable selection where logistic regression models may have convergence issues [33, 41]. However, if inferences are important to the researcher, then fitting a statistical logistic regression model would be necessary but would require an understanding of epidemiological principles (e.g., causal reasoning).

The opioid and cannabinoid models had reasonable sensitivity, specificity, area under the receiver operating characteristic curve (concordance statistic), and percent correctly classified. They had good negative predictive values but had poor positive predictive values, which is a result of rare outcomes. The practical application of our predictive models depends on how reliably they can predict positive and negative drug events in dogs. The poor positive predictive value means these models cannot be used to reliably identify a dog exposed to an opioid or cannabinoid when the exposure is unknown, as there is a high probability any positive cases the model identified are false positives. However, the high negative predictive value means these models could potentially be used to reliably predict that a dog was not poisoned by an opioid or cannabinoid. Consequently, these models could be part of the diagnostic workup to help veterinarians better advise owners concerning diagnostic and treatment options for their pets. The consequence of a false-positive may largely depend on the treatment involved in terms of cost and invasiveness. In predictive modelling, deviance ratios are also used as a statistic to measure model fit and predictive ability [39, 40, 42]. For both cannabinoid and opioid analyses, the logistic regression models seemed to outperform the lasso logistic regression models in terms of deviance ratios. However, this did not correspond to any epidemiologically meaningful differences in the models’ predictive performance.

Adjusting for autocorrelation in both, logistic and lasso logistic regression models, did not have a substantial effect on the models’ predictive abilities. This is likely because the proportion of the variance in dog opioid or cannabinoid poisoning is very small at the state level and mostly explained at the dog level. However, this may not be true for other toxicants where a greater proportion of the variance is explained at higher levels (e.g., county or state). Therefore, having the ability to only control for one level of clustering in lasso logistic regression models may limit their current utility.

### Number of variables and variables included in models

As expected, based on their purpose, the logistic regression models were more parsimonious than lasso logistic regression models and interpretable; typically, lasso is not used for inference, although there are limited methods that allow for p-values and CIs to be added by using bootstrapping for example [43, 44], and the inclusion of too many variables in logistic regression models will lead to over-fitting and convergence issues.

While controlling for autocorrelation had no major effect on our logistic regression models, it substantially reduced the number of variables in lasso logistic regression models. An inability to deal with more complex autocorrelation structures (e.g., several hierarchical levels) may be an important limitation for the performance of lasso models in some situations [40]. For instance, mixed logistic regression models, unlike lasso regression, can adjust for confounding by group and identify associations of variables measured at different hierarchical levels. The logistic regression models were very similar to the cannabinoid and opioid models made in our previous work [11, 12]. These models were mostly comprised of main effects coefficients, with a few quadratic and interaction terms. The lasso logistic regression models were comprised mainly of interaction terms. Interestingly, several of the variables which made up interaction terms did not have their main effects in the model, adding to the difficulty in the interpretation of these models and potentially increasing the number of variables to be collected for these models to be applied prospectively.

### Interpretation of variables included in the models

For cannabinoids, the dog-level variables included in the models were age, sex, and breed class. For opioids, the dog-level variables included were weight, age, sex, reproductive status, and breed class. The direction and magnitude of the odds ratios associated with dog-level predictor variables were consistent with our previous research [11, 12]. However, the disorder categories and the knowledge gaps they fill have not been explored previously. There were reduced odds of a cannabinoid or opioid poisoning event when digestive, hematopoietic, integumentary, or metabolic disorders were related to the call. With the exception of digestive disorders, these other disorders are not involved in the toxicological effects of these drugs. While constipation can result from the consumption of opioids [45], it is unlikely to be part of an acute poisoning event that results in a call to the APCC. There were greater odds of a call being related to both opioids and cannabinoids when the owner reported that the dog had cardiovascular, general, and nervous disorders. These findings were expected since both opioids and cannabinoids affect heart rate, they cause an obvious high or euphoric state, and both have strong effects on the central nervous system [46, 47]. There were greater odds of an opioid poisoning event, but a lower odds of a cannabinoid poisoning event when respiratory disorders were reported with the call. This is likely related to the potentially severe respiratory depression experienced with opioid poisonings. There were greater odds of a cannabinoid poisoning event but a lower odds of an opioid poisoning event when sensory disorders were reported. This may be due to the dilatory effect of cannabinoids. There were greater odds of cannabinoid poisoning events when urinary disorders were reported, possibly due to the incontinence caused by exposure to cannabinoids. This information may be helpful in terms of understanding what types of disorders are being observed by users of the APCC and where to focus educational resources to raise awareness of signs of acute poisoning with these drugs.

## Conclusion

As recreational drug use continues to increase in humans [1–7], it is important to explore tools to identify pet exposures when individuals are unwilling or unable to report the potential for exposure and subsequent substance ingestion by their pet. Although we found that our cannabinoid and opioid models had poor positive predictive values, we could use these models to help build diagnostic evidence to reduce concerns of exposure to cannabinoids and opioids which would help veterinarians better advise clients on diagnostic and treatment plans for their pet. While lasso regression models are easier to automate and construct without epidemiological knowledge, in this study they had the same predictive abilities as logistic regression models built using epidemiological principles. Logistic regression models also have the benefit of parsimony and interpretability. While controlling for autocorrelation at the state level did not have a major effect on the predictive abilities of the models examined, controlling for autocorrelation substantially decreased the number of variables in the lasso models, suggesting that further effort to develop methods to control for autocorrelated data with lasso regression is warranted. Using logistic and multi-level logistic regression models, we were able to identify disorder categories that are associated with these acute poisoning incidents. This information may be critical in developing tools for clinicians and the public to recognize acute intoxications with these drugs in pet dogs.

## Supporting information

S1 Fig Predicted probability of a poisoning call to the APCC^a^ being related to a cannabinoid in US dogs (2005–2014), plotted against dog age. (TIF)

S2 Fig Predicted probability of a poisoning call to the APCC^a^ being related to an opioid in US dogs across different ages plotted against dog weight (2005–2014). (TIF)

S1 Table Coefficients included in a lasso logistic regression model used to predict a dog poisoning call to the APCC^a^ being related to cannabinoids (2005–2014). (DOCX)

S2 Table Coefficients included in a cluster-adjusted lasso logistic regression model used to predict a dog poisoning call to the APCC^a^ being related to cannabinoids (2005–2014). (DOCX)

S3 Table Coefficients included in a lasso logistic regression model used to predict a dog poisoning call to the APCC^a^ being related to opioids (2005–2014). (DOCX)

S4 Table Coefficients included in a cluster-adjusted lasso logistic regression model used to predict a dog poisoning call to the APCC^a^ being related to opioids (2005–2014). (DOCX)
