# Supplementary figures and images for: Comparing the diagnostic performance of ordinary, mixed, and lasso logistic regression models at identifying opioid and cannabinoid poisoning in U.S. dogs using pet demographic and clinical data reported to an animal poison control center (2005–2014)
Source: PLoS One. 2023 Jul 10;18(7):e0288339. doi: 10.1371/journal.pone.0288339 (PMC10332589; doi:10.1371/journal.pone.0288339)

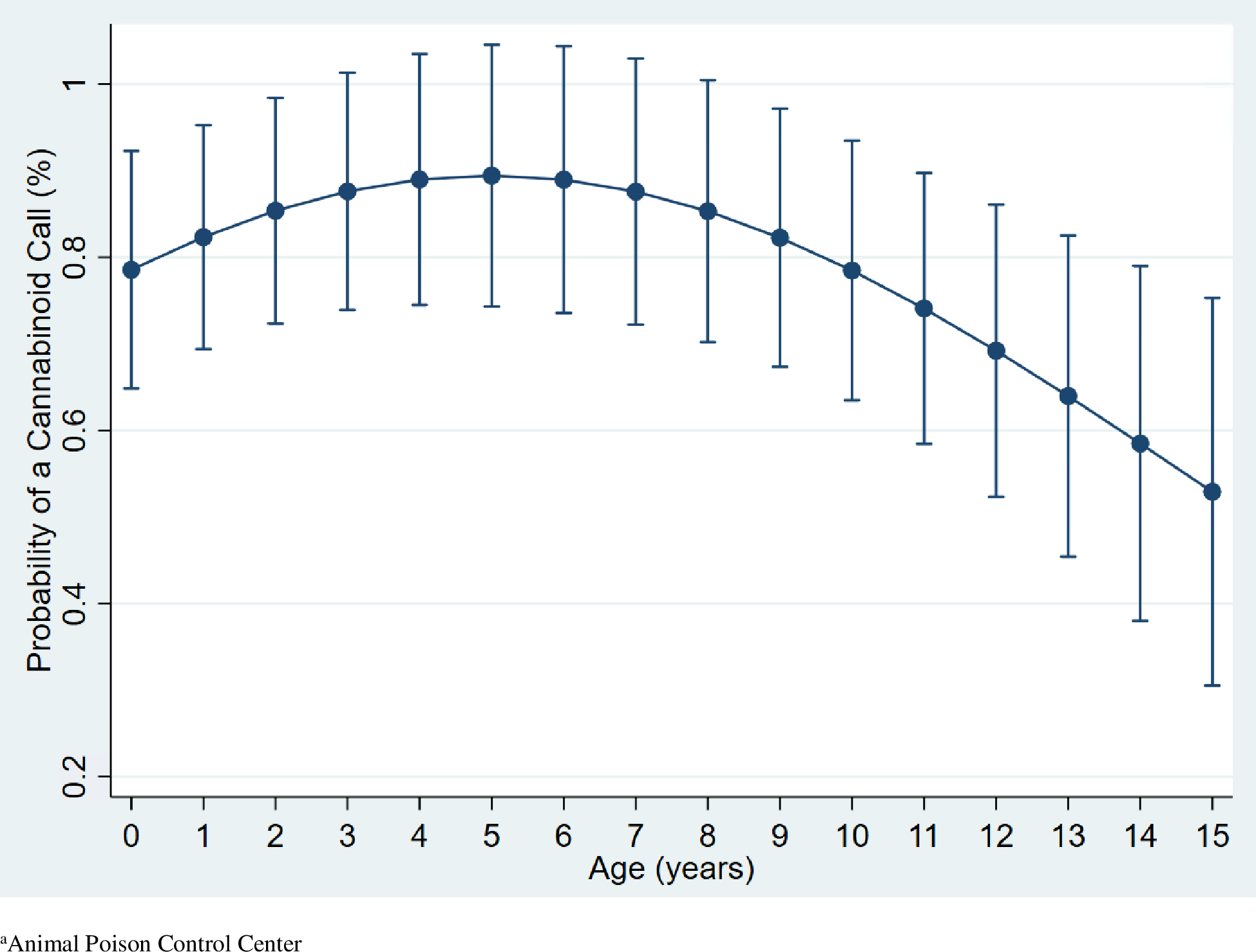

Supplement: S1 Fig — (TIF) [file pone.0288339.s001.tif]

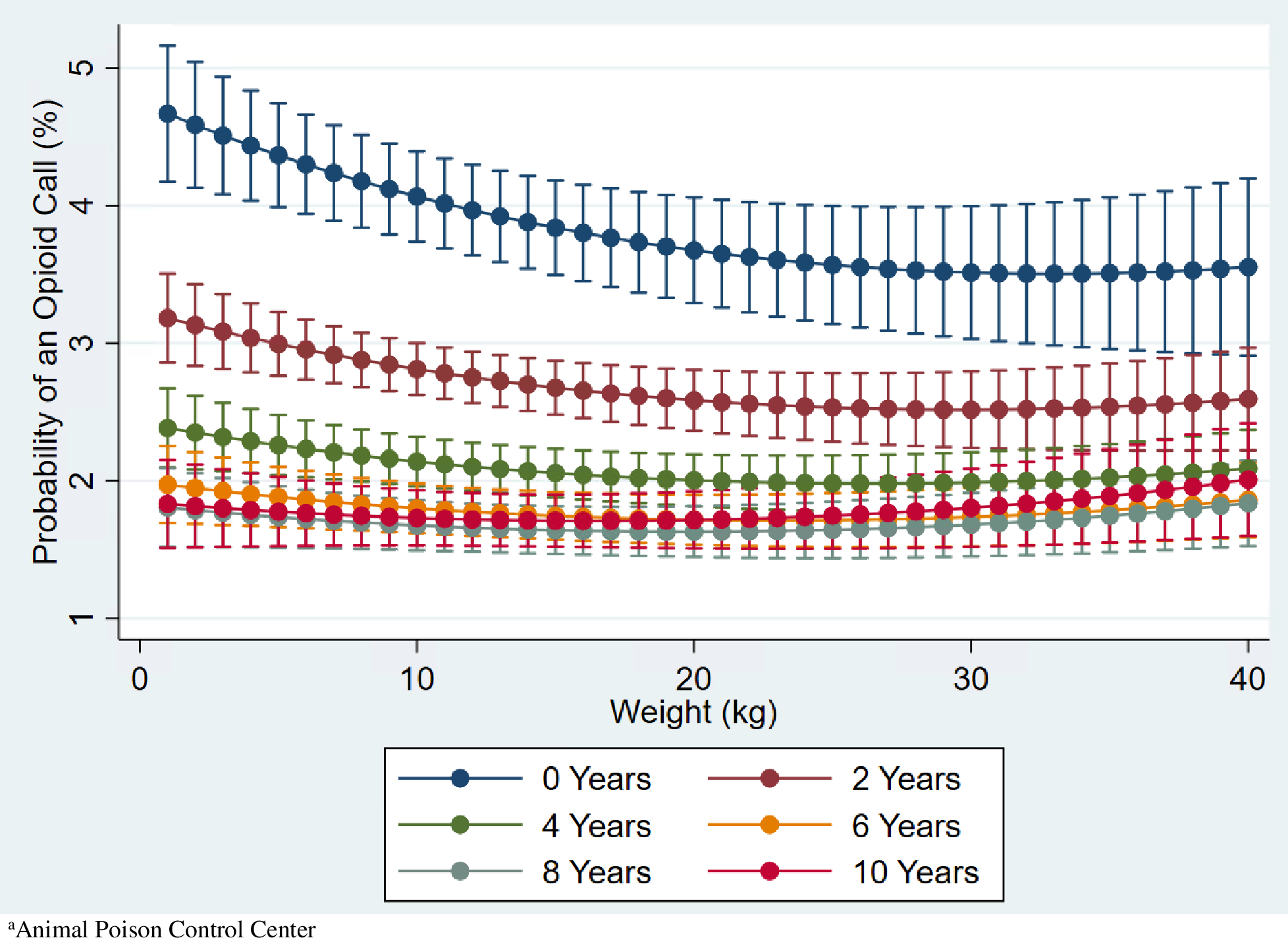

Supplement: S2 Fig — (TIF) [file pone.0288339.s002.tif]
