# Supplementary material for: Comparing the diagnostic performance of ordinary, mixed, and lasso logistic regression models at identifying opioid and cannabinoid poisoning in U.S. dogs using pet demographic and clinical data reported to an animal poison control center (2005–2014)
Source: PLoS One. 2023 Jul 10;18(7):e0288339. doi: 10.1371/journal.pone.0288339 (PMC10332589; doi:10.1371/journal.pone.0288339)
Supplement: S1 Table — (DOCX) [file pone.0288339.s003.docx]

**S1 Table - Coefficients included in a lasso logistic regression model used to predict a dog poisoning call to the APCC^a^ being related to cannabinoids (2005-2014).**

| **Parameter** | **Coefficient** |
| --- | --- |
| Age^2^ | -0.0365 |
| Weight | -0.0325 |
| Sporting | -0.00507 |
| Digestive | -0.205 |
| General | 0.0622 |
| Integumentary | -0.109 |
| Metabolic | -0.0609 |
| Nervous | 1.206 |
| Sensory | 0.0520 |
| Unknown Sex x Age | 0.0143 |
| Toy x Age | -0.0255 |
| Nervous x Age | 0.0390 |
| Sensory x Age | 0.0360 |
| Urinary x Age | -0.0733 |
| Musculoskeletal x Age | -0.00447 |
| Male x Weight | -0.00415 |
| Sporting x Weight | -0.00896 |
| Terrier x Weight | 0.00226 |
| Neutered x Weight | -0.0414 |
| Behavioural x Weight | 0.0565 |
| Digestive x Weight | -0.100 |
| Metabolic x Weight | -0.0471 |
| Urinary x Weight | 0.104 |
| Non-Sporting x Male | 0.0231 |
| Sporting x Male | -0.00903 |
| Sporting x Unknown Sex | 0.0215 |
| Terrier x Unknown Sex | 0.0237 |
| Unknown Reproductive Status x Male | -0.0205 |
| Unknown Reproductive Status x Unknown Sex | -0.0482 |
| Cardiovascular x Male | 0.0254 |
| Cardiovascular x Unknown Sex | -0.00271 |
| General x Male | 0.00809 |
| General x Unknown Sex | -0.0137 |
| Nervous x Male | 0.0761 |
| Sensory x Male | -0.0432 |
| Urinary x Male | 0.00950 |
| Neutered x Non-Sporting | 0.0143 |
| Unknown Reproductive Status x Sporting | -0.00997 |
| Unknown Reproductive Status x Toy | -0.0211 |
| Behavioural x FSS, Misc & Other | 0.0190 |
| Behavioural x Sporting | -0.00242 |
| Behavioural x Terrier | 0.0191 |
| Behavioural x Toy | -0.0176 |
| Digestive x Non-Sporting | -0.0105 |
| Digestive x Sporting | -0.0881 |
| Digestive x Working | -0.0197 |
| Cardiovascular x Hound | -0.0424 |
| Cardiovascular x FSS, Misc & Other | -0.00177 |
| Cardiovascular x Non-Sporting | 0.0140 |
| Cardiovascular x Terrier | 0.00566 |
| Cardiovascular x Toy | 0.0134 |
| General x Hound | -0.00581 |
| General x Non-Sporting | 0.0313 |
| Hematopoietic x FSS, Misc & Other | 0.0159 |
| Hematopoietic x Non-Sporting | -0.00793 |
| Metabolic x Hound | -0.0314 |
| Metabolic x Sporting | -0.0337 |
| Nervous x Toy | 0.0845 |
| Neutered x FSS, Misc & Other | -0.00691 |
| Neutered x Toy | -0.100 |
| Sensory x FSS, Misc & Other | 0.0186 |
| Sensory x Non-Sporting | 0.0318 |
| Sensory x Terrier | 0.0217 |
| Sensory x Toy | 0.0419 |
| Sensory x Working | -0.0128 |
| Urinary x FSS, Misc & Other | 0.0187 |
| Urinary x Non-Sporting | -0.0230 |
| Urinary x Terrier | 0.00927 |
| Urinary x Toy | -0.0632 |
| Urinary x Working | -0.00571 |
| Musculoskeletal x FSS, Misc & Other | -0.0432 |
| Musculoskeletal x Terrier | 0.0301 |
| Musculoskeletal x Toy | 0.0429 |
| Digestive x Neutered | -0.130 |
| Digestive x Unknown Reproductive Status | -0.00764 |
| Cardiovascular x Neutered | 0.0635 |
| Cardiovascular x Unknown Reproductive Status | 0.0192 |
| General x Unknown Reproductive Status | 0.0601 |
| Hematopoietic x Neutered | -0.0412 |
| Hematopoietic x Unknown Reproductive Status | -0.00022 |
| Nervous x Neutered | 0.0238 |
| Respiratory x Unknown Reproductive Status | -0.0120 |
| Sensory x Neutered | 0.0117 |
| Sensory x Unknown Reproductive Status | -0.00557 |
| Urinary x Neutered | 0.00684 |
| Urinary x Unknown Reproductive Status | -0.00657 |
| Digestive x Behavioural | 0.0203 |
| Cardiovascular x Behavioural | -0.0687 |
| Respiratory x Behavioural | -0.0637 |
| Sensory x Behavioural | -0.00703 |
| Urinary x Behavioural | -0.0207 |
| Musculoskeletal x Behavioural | 0.0182 |
| Metabolic x Digestive | -0.0304 |
| Respiratory x Digestive | -0.0110 |
| Sensory x Digestive | 0.0646 |
| Urinary x Digestive | -0.0919 |
| General x Cardiovascular | 0.0178 |
| Nervous x Cardiovascular | 0.0373 |
| Sensory x Cardiovascular | 0.00542 |
| Musculoskeletal x Cardiovascular | 0.0220 |
| Hematopoietic x General | -0.00828 |
| Sensory x General | -0.0243 |
| Nervous x Hematopoietic | -0.0791 |
| Sensory x Hematopoietic | 0.00712 |
| Musculoskeletal x Hematopoietic | -0.00855 |
| Nervous x Integumentary | -0.0853 |
| Urinary x Integumentary | 0.0133 |
| Nervous x Lymphatic | -0.00341 |
| Neutered x Nervous | -0.00422 |
| Respiratory x Nervous | -0.220 |
| Urinary x Nervous | 0.211 |
| Traumatic x Nervous | -0.00670 |
| Musculoskeletal x Nervous | -0.0489 |
| Sensory x Reproductive | -0.00076 |
| Sensory x Respiratory | -0.0131 |
| Urinary x Sensory | 0.0174 |
| Musculoskeletal x Sensory | 0.00797 |

^a^Animal Poison Control Center
